# Synergy of Omeprazole and Praziquantel *In Vitro* Treatment against *Schistosoma mansoni* Adult Worms

**DOI:** 10.1371/journal.pntd.0004086

**Published:** 2015-09-24

**Authors:** Giulliana T. Almeida, Regina C. G. Lage, Leticia Anderson, Thiago M. Venancio, Helder I. Nakaya, Patrícia A. Miyasato, Henrique K. Rofatto, Adhemar Zerlotini, Eliana Nakano, Guilherme Oliveira, Sergio Verjovski-Almeida

**Affiliations:** 1 Departamento de Bioquímica, Instituto de Química, Universidade de São Paulo, São Paulo, Brazil; 2 Genomics and Computational Biology Group, Centro de Pesquisas René Rachou - FIOCRUZ, Belo Horizonte, Minas Gerais, Brazil; 3 Instituto Butantan, São Paulo, São Paulo, Brazil; 4 Vale Technology Institute, Belém, Pará, Brazil; University of Pennsylvania, UNITED STATES

## Abstract

**Background:**

Treatment and morbidity control of schistosomiasis relies on a single drug, praziquantel (PZQ), and the selection of resistant worms under repeated treatment is a concern. Therefore, there is a pressing need to understand the molecular effects of PZQ on schistosomes and to investigate alternative or synergistic drugs against schistosomiasis.

**Methodology:**

We used a custom-designed *Schistosoma mansoni* expression microarray to explore the effects of sublethal doses of PZQ on large-scale gene expression of adult paired males and females and unpaired mature females. We also assessed the efficacy of PZQ, omeprazole (OMP) or their combination against *S*. *mansoni* adult worms with a survival *in vitro* assay.

**Principal Findings:**

We identified sets of genes that were affected by PZQ in paired and unpaired mature females, however with opposite gene expression patterns (up-regulated in paired and down-regulated in unpaired mature females), indicating that PZQ effects are heavily influenced by the mating status. We also identified genes that were similarly affected by PZQ in males and females. Functional analyses of gene interaction networks were performed with parasite genes that were differentially expressed upon PZQ treatment, searching for proteins encoded by these genes whose human homologs are targets of different drugs used for other diseases. Based on these results, OMP, a widely prescribed proton pump inhibitor known to target the ATP1A2 gene product, was chosen and tested. Sublethal doses of PZQ combined with OMP significantly increased worm mortality *in vitro* when compared with PZQ or OMP alone, thus evidencing a synergistic effect.

**Conclusions:**

Functional analysis of gene interaction networks is an important approach that can point to possible novel synergistic drug candidates. We demonstrated the potential of this strategy by showing that PZQ in combination with OMP displayed increased efficiency against *S*. *mansoni* adult worms *in vitro* when compared with either drug alone.

## Author Summary

Schistosomiasis causes severe health problems in endemic areas of Africa, Southeast Asia, and Central and South America. Praziquantel is the drug of choice for treatment of at-risk populations; however, evolution of resistant worms under repeated treatment is of great concern. Combining praziquantel with another drug could not only increase efficacy of praziquantel, but also eventually hamper development of drug resistance. Our study reports the global praziquantel-induced transcriptional changes of *Schistosoma mansoni* adult worms *in vitro*, in the context of the mature female mating status (paired or unpaired). We identified sets of genes that were differentially affected in paired or unpaired mature females; we also identified genes that were similarly affected in males and females. Aiming to find possible new candidates to be tested as synergistic drugs, we used functional analysis of gene interaction networks to identify parasite genes whose expression was affected by praziquantel, and encode proteins whose human homologs are targets of different drugs already used to treat other diseases. This analysis suggested omeprazole, a widely prescribed drug, as a potential partner for praziquantel in a combination treatment. Finally, we demonstrated that this praziquantel-omeprazole combination resulted in increased worm lethality *in vitro* when compared with praziquantel or omeprazole alone.

## Introduction

Schistosomiasis is a worldwide neglected disease that kills over 200,000 people annually. The disease is endemic in 76 countries distributed throughout Africa, Southeast Asia, and Central and South America, with more than 230 million infected people. *Schistosoma mansoni* is the most widespread causative species and the only one that constitutes a health problem in the Americas [1].

Until now, there is no effective vaccine available for schistosomiasis, and praziquantel (PZQ) is the only drug of choice for large-scale treatment of at-risk populations. PZQ is highly effective against all *Schistosoma* species that infect humans, having a high cure rate, low toxicity, and low cost [2,3]. However, PZQ treatment does not prevent reinfection, and infected animal models have shown resistance to PZQ upon repeated treatment [4,5]. The mechanisms of action of PZQ that result in the disruption of Ca^++^ homeostasis in the parasite, with paralysis and tegumental disruption, as well as the mechanism involved with parasite resistance to PZQ, have been reviewed [6,7].

The susceptibility of schistosomes to PZQ is dependent on the stage of worm maturation, young parasites being sensitivity only after 3–4 weeks of infection [8]. In mixed-sex infection, when male worms only are considered, they are more sensitive to PZQ than the whole (male + female) population [9], thus creating the possibility that paired mature females could be left unpaired after the death of their male partners [9]. On the other hand, in single-sex infection the unpaired females cannot fully mature [10–12], and are less sensitive to PZQ than the mature female from mixed-sex infection [9]. Given that pairing regulates the female-specific gene expression [13], there is a possibility that unpaired mature females could be more resistant to PZQ than paired mature females. The consequences of such possible differential susceptibility on the pattern of gene expression changes induced by PZQ have never been evaluated.

In this work, we analyzed the global PZQ-driven transcriptional changes of *S*. *mansoni* mature female and male worms in the context of the female mating status (paired or unpaired). Using our custom-designed *S*. *mansoni* microarray [14] we identified sets of genes that were affected in both paired and unpaired mature females, however with opposite gene expression patterns (up-regulated in paired and down-regulated in unpaired mature females), indicating that the transcriptional changes induced by PZQ in mature females are heavily influenced by the mating status. With functional analysis of gene interaction networks it was possible to identify a Na/K-ATPase gene differentially expressed in the presence of PZQ, whose human homolog gene product is a target of omeprazole (OMP), a drug used in humans for treating gastric ulcer, thus suggesting that OMP could be tested as a synergic drug against schistosomes. Indeed, we found that PZQ and OMP combinations were synergistic in *a*dult worms when tested *in vitro*, with OMP enhancing the effects of sublethal doses of PZQ.

## Methods

### Parasite materials

*S*. *mansoni* (LE strain) was maintained at *Centro de Pesquisas René Rachou*, *Fiocruz (Brazil)* by routine passage through *Biomphalaria glabrata* snails and female Swiss mice (mean weight 25 g), which were infected by subcutaneous route with 100 ± 10 cercariae [15]. Seven weeks after the infection, adult worms were recovered by perfusion of the portal-mesenteric system with 0.85% saline solution and 1% heparin [16].

Male and female adult worms obtained from the perfusion of mice were recovered mostly as paired mature couples, were washed in RPMI medium and were either kept as paired couples (“paired females” and “paired males”). Alternatively, mature females were separated from males by incubation at room temperature for 20 min and subsequently identified as “unpaired mature females”. Unpaired mature females and paired males-females were placed in separate plastic dishes containing RPMI 1640 medium (Gibco) supplemented with 1% Hepes (Gibco) pH 7.4, 10% Fetal Bovine Serum (FBS), 100 μg/mL Penicillin/ Streptomycin and 0.5 μg/mL Amphotericin B (Sigma). Worms were incubated at 37°C and 5% CO_2_ overnight to recover from the eventual stress of perfusion and separation, and in the following morning 0.1μg/ml PZQ (Far-Manguinhos, number 06082207) in 1% DMSO was added and further incubation proceeded for 16 h. In parallel, controls were incubated overnight under the same conditions, and in the following morning the vehicle (1% DMSO) was added and further incubation proceeded for 16 h. After the 16 hours, treated and control worms were washed in a RPMI 1640 medium without a pH indicator; after a 30-minute waiting period, parasites were observed and the few worms that were contracted or had acquired an opaque appearance were considered dead and discarded. The remaining treated or control worms were separately collected into RNA—later solution (Ambion), separated by gender (in the case of paired worms) and placed at 4°C overnight until RNA extraction. The experiment was performed on two biological replicates.

### Total RNA extraction and microarray experiments

Total RNA was extracted using Trizol (Invitrogen) according to the manufacturer's instructions. RNA was quantified using the Nanodrop ND-1000 UV/Vis spectrophotometer and RNA integrity was checked with Agilent 2100 Bioanalyzer microfluidic electrophoresis. Total RNA (300 ng) from each sample was converted to cDNA, linearly amplified with T7 RNA polymerase, and labeled using the Low RNA Input Fluorescent Linear Amplification Kit (Agilent Technologies) following manufacturer specifications. Dye swap was used in the technical replicate to account for dye biases in the microarray experiments. Two technical replicate experiments were performed for each of two biological replicates.

The custom-designed oligonucleotide microarray used in this work was previously described [14] and it is comprised of 60-mers that probe the two strands of each contig that was assembled in the *S*. *mansoni* transcriptome project [17], including 19,829 probes representing 7,307 unique Smp genes predicted in the parasite genome v.5.2 [18] and 5,717 probes for additional *S*. *mansoni* transcripts [17]. The array slides (4x44K) were manufactured by Agilent Technologies. The microarray platform design along with gene name annotations was deposited at NCBI Gene Expression Omnibus (GEO) under accession number GPL8606. Slides were washed and processed according to the Agilent 60-mer Oligo Microarray Processing Protocol (Agilent Technologies) and scanned on a GenePix 4000B scanner (Molecular Devices).

### Microarray analysis

Intensity data were extracted from the scanned images with Feature Extraction software, version 11.0.1.1 (Agilent Technologies) with the standard two-color LOWESS normalization correction method. Microarray data are deposited at GEO (GSE66697).

For a gene that was represented in the array by multiple probes, we chose a single representative probe by selecting the probe with the smallest coefficient of variation (CV) among the replicas of almost all previously published experiments using this array; the selected probes (marked "YES, to be used in expression analysis") are listed in S1 Table along with gene names and annotations. A total of 11,132 probes marked “YES, to be used in expression analysis” represent each of 11,132 unique protein-coding genes under analysis. Additionally, there are 8,065 probes also marked “YES, to be used in expression analysis” having a sequence complementary to that of each of 8,065 protein-coding Smp genes, thus probing the eventual cis-antisense transcripts. The signal from these probes (marked “antisense” in S1 Table) was included in differential expression analysis, but excluded from further functional analyses. The calculated Pearson correlation of probes signal intensities between biological samples in the microarray experiments was: for paired males r = 0.94 ± 0.02, for paired females r = 0.88 ± 0.02, and for unpaired mature females r = 0.87 ± 0.03.

The Significance Analysis of Microarrays (SAM) tool was used to identify differentially expressed genes [19], applying the SAM one-class test, and genes were considered as significantly differentially expressed at q-value ≤ 0.05. Finally, we applied a cutoff filter and kept as differentially expressed only those genes with an average fold change greater than 1.5. In order to identify the differentially expressed genes upon PZQ exposure, for each tested condition the statistical analysis was performed with the replicate values of the ratio between gene expression in the PZQ-treated sample and in the corresponding no-drug (1% DMSO vehicle) control sample. The lists of significantly differentially expressed genes detected in each condition (unpaired mature females vs. its control, paired females vs. its control, and paired males vs. its control) were subsequently compared against each other.

### Pathway analysis of the differentially expressed genes

Ingenuity Pathway Analysis software (IPA) was used for transcriptomic analyses, identifying significantly enriched gene networks among the differentially expressed *S*. *mansoni* genes. IPA offers a systems-perspective for gene expression changes by integrating available literature information on model organisms (human, mouse and rat) regarding molecular and chemical interactions, cellular phenotypes as well as about signaling and metabolic pathways. *S*. *mansoni* genes encoding putative homologues of human proteins were identified as follows: the predicted *S*. *mansoni* Smp protein full-length sequence corresponding to each unique probe was aligned to the GenBank human proteins dataset (using BLASTp), with a BLASTp cutoff e-value ≤1×10^−9^ and query coverage ≥ 60%, thus identifying and annotating a total of 3,779 unique probes on the oligo-microarray. The GI number of each putative human homolog was associated to the respective *S*. *mansoni* probe (S2 Table) and the array expression and statistical significance analyses results were uploaded to IPA version 7.6.

Our oligo-microarray contains probes for transcribed regions that do not correspond to *S*. *mansoni* genes predicted in the genome v.5.2 [18]. In such cases we used the contig sequence obtained in the transcriptome project [17] to search for homologs in *S*. *japonicum* or other species, as well as a putative human homolog with a predicted function. We found a human homolog for 90% of the differentially expressed transcript (S2 Table), and we therefore included them in the functional analyses described above.

### Real-time RT-PCR

Random-primed reverse transcription (RT) was performed using 1.5 μg of total RNA according to the Super Script III kit protocol (Invitrogen). The relative transcriptional levels were determined through quantitative PCR (qPCR) (primers are listed in S3 Table) with Sybr Green PCR Master Mix (Applied Biosystem) using the 7500 Real-Time PCR System (Applied Biosystem). Real time data was normalized in relation to the level of expression of the housekeeping gene PSMD4 (Smp_000740) according to [20]; in fact, PSMD4 was stable and it was not detected as differentially expressed in the microarray experiments. *P-value* was determined for the two biological replicates of the RT-qPCR assay with Student’s *t*-test, using one tail distribution and homoscedastic variance.

### *In vitro* survival experiments with *S*. *mansoni*

*S*. *mansoni* (BH strain) adult worms harvested from hamsters were washed in RPMI 1640 medium (Gibco) supplemented with 200 μg/ml of streptomycin, 200 IU/ml of penicillin (Invitrogen), and 25 mM of Hepes. For each of two biological replicates, six different sets of twenty adult worm pairs each (120 worm pairs) were incubated in 24-well culture plates; each well contained a worm pair in 1 ml of the above medium supplemented with 10% heat-inactivated calf serum at 37°C in a 5% CO_2_ atmosphere. The sublethal dose to be used for these survival assays was determined with a dose-response curve; the LD5 at 24 h incubation was obtained and used in the subsequent survival assays, namely 150 ng/mL PZQ when scoring male survival only, and 532 ng/mL when scoring female survival only. Thus, PZQ and/or OMP were added to the culture under the following five different conditions: 25 μg/mL OMP; 150 ng/mL PZQ; 25 μg/mL OMP + 150 ng/mL PZQ; 532 ng/mL PZQ; 25 μg/mL OMP + 532 ng/mL PZQ; and 1.5% DMSO was added to the sixth set as a negative control. Loss of worm motor activity, and acquisition of an opaque appearance were used as criteria for “death” of the parasites, which were monitored after 2, 24, 48, 72, 96 and 120 h using an inverted microscope. For accessing the effect of the drugs on males, male survival data from the 25 μg/mL OMP, the 150 ng/mL PZQ and the 25 μg/mL OMP + 150 ng/mL PZQ conditions were used. For the effect on females, female survival data from the 25 μg/mL OMP, the 532 ng/mL PZQ and the 25 μg/mL OMP + 532 ng/mL PZQ conditions were used. The statistical significance of the synergistic effect of PZQ+OMP compared with PZQ alone, acting either on males or females, was estimated with the Kaplan-Meier survival function (Log-rank Mantel-Cox test, Prism 6 software, GraphPad) using all the survival/death events of the two biological replicates (n = 40) for each condition.

For schistosomula preparation, cercariae were shed from infected snails and subsequently converted to schistosomula by mechanical transformation, using a Vortex mixer as described by Ramalho-Pinto and co-workers [21]. Schistosomula were cultivated in 169 medium containing antibiotics and supplemented with 2% fetal bovine serum at 37°C in a 5% CO_2_ atmosphere [22]. For *in vitro* survival assays, 3-hour old schistosomula were used. After the culture period, these schistosomula were transferred to 96-well culture microplates (approximately 300 parasites per well into 49 different wells) and maintained in culture (37°C, 5% CO_2_) in 169 medium containing PZQ and/or OMP under seven different conditions with the final volume of 100 μl per well: 25 μg/mL OMP; 150 ng/mL PZQ; 532 ng/mL PZQ; 25 μg/mL OMP + 150 ng/mL PZQ; 25 μg/mL OMP + 532 ng/mL PZQ; 1.5% DMSO as the negative control; and 10 μg/ml PZQ as the positive control.

The culture plates were monitored for viability of schistosomula according to Peak et al. [23] by adding 2 μg/mL propidium iodide (PI) (Sigma-Aldrich) and 0.5 μg/mL fluorescein diacetate (FDA) (Life Technologies) on a daily basis for 7 days, and observing the parasites under light and fluorescence microscopy. Under light microscopy, viable parasites were scored by preserved mobility and lack of opacity. Under fluorescence microscopy, schistosomula death was scored by a red fluorescence signal (572 nm emission microscope filter); schistosomula living cells convert FDA into charged fluorescein by parasite esterase activity, staining the schistosomula with a green fluorescence signal (492 nm emission microscope filter) [23]. On each observation day, the fluorescent stains were added to one of the seven wells of each of the seven conditions, the percent of survival was recorded, and the stained cultures were discarded. Two replicate biological experiments were performed, and a typical result is shown.

### Ethics statement

*In vivo* studies were conducted in compliance with the guidelines of the Brazilian Collegiate of Animal Experimentation (COBEA) and approved by the Commission on the Ethical Use of Animals (CEUA-FIOCRUZ) protocol number L-018/09 approved on Jan. 23, 2009.

## Results and Discussion

### Transcriptional changes driven by PZQ in *S*. *mansoni* paired and unpaired mature females

The experiments described below compare the transcriptomic profiles of paired with unpaired mature females treated with 0.1 μg/ml PZQ *in vitro*. The unpaired mature females cohort was derived from a mixed-sex infection; they were separated from males immediately after perfusion and kept separated overnight before PZQ was added (see Methods). This cohort is distinct from unpaired females obtained from single-sex infections, once the influence of the male on the female gene transcription [13] had already taken place during mating. The idea was that this cohort could in part mimic the following *in vivo* situation: it is known that males are more sensitive to PZQ than the whole (male + female) population [9], thus creating the possibility that paired mature females could be left unpaired after the death of their male partners [9]. It is noteworthy that Pica-Mattoccia and Cioli have demonstrated that unpaired single-sex females are also less sensitive to PZQ than males [9]. As the pattern of gene expression changes induced by PZQ in unpaired females has never been evaluated before, we decided to analyze the unpaired mature females, comparing them with no-drug controls and with paired ones.

In selecting the treatment conditions for transcriptome analysis, we chose to use a sublethal dose of 0.1 μg/ml PZQ for 16 hours [9], which would induce transcriptional changes without predominantly triggering the death pathways.

The total number of genes detected as differentially expressed (q-value < 0.05) in the presence of PZQ compared with the no-drug control was almost 3 times lower in paired than in unpaired mature females (486 and 1,434 genes, respectively) (Table 1). The lists of differentially expressed genes are given in S4 Table. Moreover, there is a tendency for gene up-regulation in PZQ-treated paired females (476 up-regulated genes, or 98% of all affected genes in paired females when compared with the no-drug control), whereas down-regulation predominates in the PZQ-treated unpaired mature females (1,109 down-regulated genes, or 77% of all affected genes in unpaired mature females when compared with the no-drug control).

**Table 1:** General information about gene expression of paired or unpaired mature females exposed to PZQ compared with no-drug controls.

*S*. *mansoni* genes	Expressed genes ^a^	Paired Females			Unpaired Mature Females		
		Diff. expressed ^b^	Down-regulated	Up-regulated	Diff. expressed ^b^	Down-regulated	Up-regulated
Detected on the microarrays	8,180	486	10	476	1,434	1,109	325
Predicted in the genome	5,903	351	4	347	1,005	809	196
Not predicted in the genome^c^	2,277	135	6	129	429	300	129

^a^ Number of genes expressed in at least one condition, *i*.*e*. in no-drug control or in treated worms of either the paired or the unpaired worm groups.

^b^ q-value < 5%

^c^ Either predicted in *S*. *japonicum*, present in other species, or detected only in *S*. *mansoni* without close homologs in other species (no match).

It is interesting to note that 2,277 transcripts that were detected in the arrays as expressed by *S*. *mansoni* are not predicted in the parasite genome sequence (Table 1), corresponding to 28% of all expressed transcripts. Similar proportions (28 to 30%) of transcripts not predicted in the genome were detected as differentially expressed under PZQ treatment of paired (135 out of 486, or 28%) and unpaired mature females (429 out of 1,109, or 30%) when compared with the no-drug control (Table 1) (listed in S4 Table). These potentially novel genes or gene fragments deserve future attention.

Next, we looked for the overlap within the lists of affected genes and found that 219 of them were simultaneously affected by PZQ both in paired and unpaired mature females (Fig A (upper panel) in S1 Text). Notably, 96% of these genes (210 out of 219) showed an opposite gene expression pattern depending on the pairing status of the females, and again the majority (206 out of 210) were up-regulated in paired and down-regulated in unpaired mature females (Fig A (lower panel) in S1 Text). These genes are listed in S4 Table.

**Fig 1:**
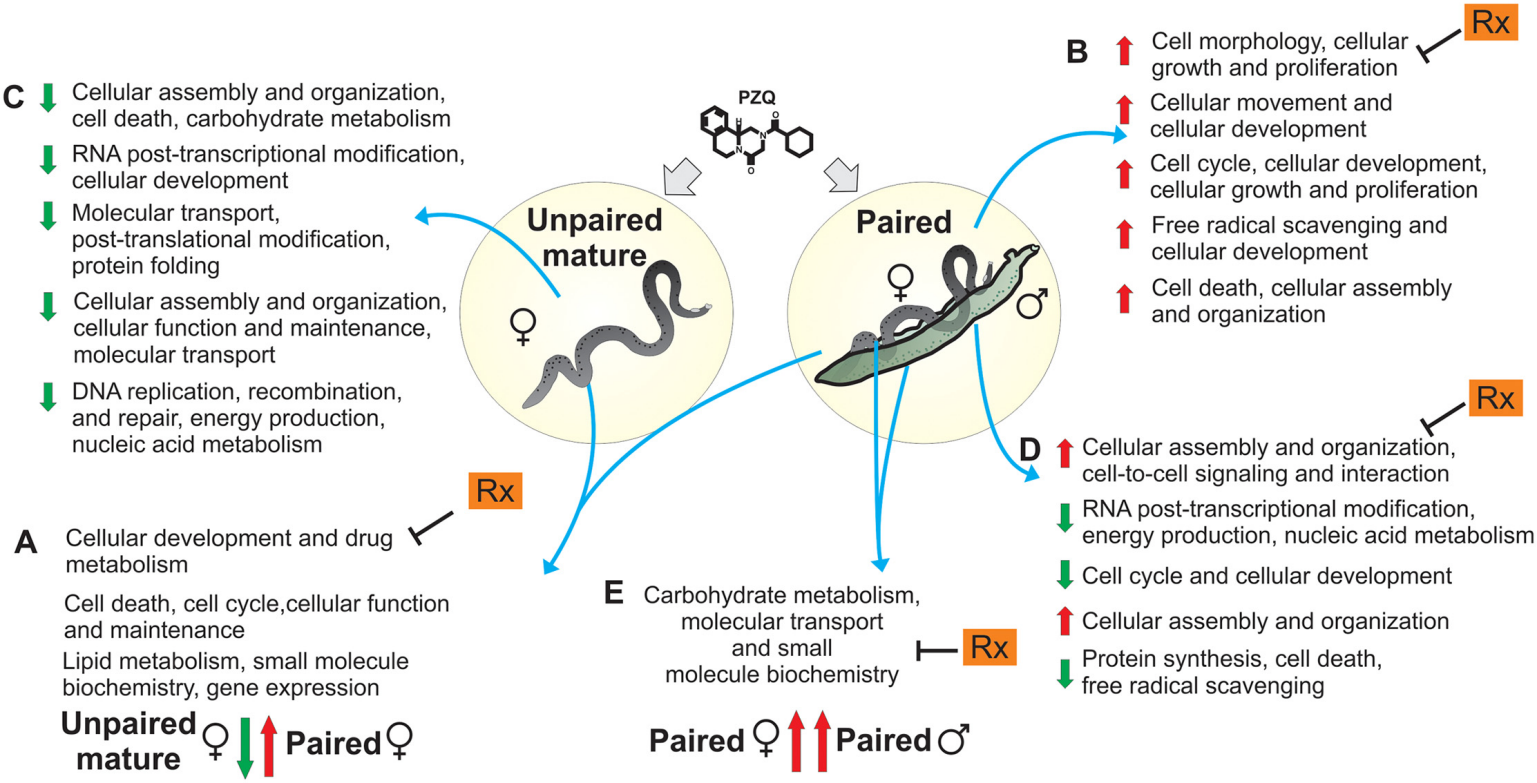
Global transcriptional changes driven by PZQ on *S*. *mansoni* adult worms. Overall summary with the names of the associated functional interaction networks identified as significantly enriched with differentially expressed *S*. *mansoni* genes in different comparisons: (A) genes affected by PZQ in opposite directions in paired or unpaired mature females, (B) all genes affected by PZQ in paired females, (C) all genes affected by PZQ in unpaired mature females, (D) all genes affected by PZQ in paired males, (E) genes affected in common in paired males and females. Color arrows indicated the direction of change in expression of the majority of the genes in each network: green implies a down-regulated expression of the majority of the genes in PZQ-treated worms, and red an up-regulated expression of the majority of the genes in PZQ-treated worms when compared with their respective no-drug controls. Rx corresponds to the identification of new candidate drugs known to act on the encoded protein products of the homolog human genes present in the indicated network, as described in the main text.

Functional analyses of these differentially expressed genes were carried out with the IPA tool, aiming to find significantly enriched gene interaction networks comprised of affected genes whose products are known targets of drugs already used in humans for other disease conditions. Among the 210 differentially expressed genes affected by PZQ in opposite directions in paired and unpaired mature females, IPA found 3 gene interaction networks (Fig 1A) that were statistically enriched (p-values from 10^−52^ to 10^−28^) with 56 genes that were affected by PZQ in opposite directions. The first most significantly enriched network (p-value = 10^−52^) is related to cellular development and differentiation and drug metabolism. The second network (p-value = 10^−41^) is associated with cell death, cell cycle, cellular function and maintenance; the third network (p-value = 10^−28^) is related to lipid metabolism, small molecule biochemistry and gene expression. (See S5 Table for the list of genes in the networks).

Although no clinically relevant resistance to PZQ has been documented thus far, reliance on a single drug is a risky endeavor [24,25], so it is imperative to explore new drug therapies, possibly including combination therapies [26]. We therefore looked, among the enriched networks, for genes whose encoded human protein homologs are known targets of drugs that are used against other diseases. The idea was to point to candidates to be tested as possible synergistic drugs in combination with PZQ against parasites *in vitro* and *in vivo* in the mouse model.

**Fig 2:**
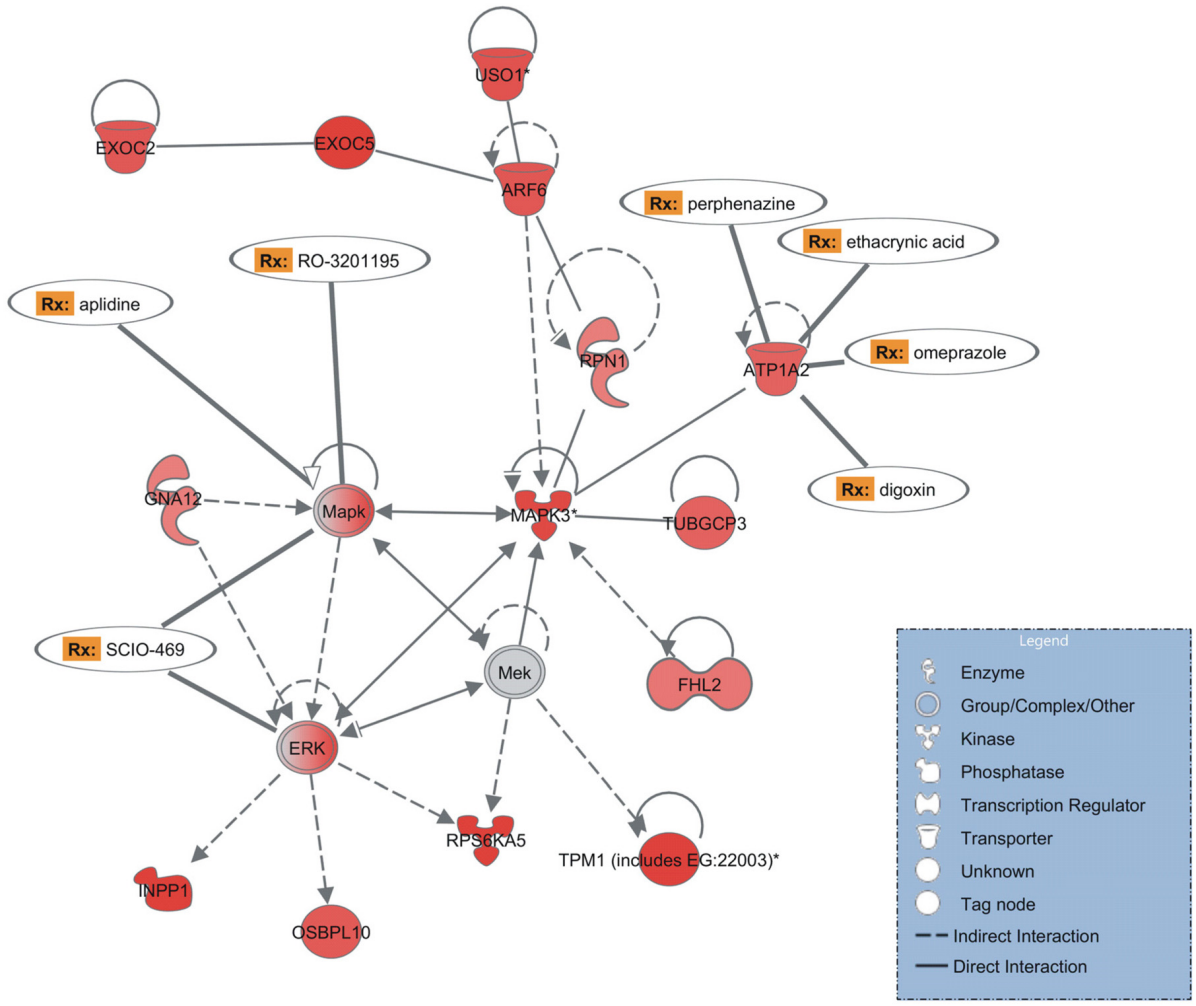
Enriched gene interaction network detected with opposite expression patterns in PZQ-treated paired or unpaired mature females. This gene interaction network is significantly enriched (p-value = 10^−52^) with differentially expressed genes related to cellular development and drug metabolism whose expression was affected by PZQ in paired as well as in unpaired mature females (with opposite patterns). The shapes of elements correspond to different types of molecules, as indicated in the inset box. Arrows indicate the relationship between the elements: dashed or solid lines indicate indirect or direct interactions, respectively. The color intensity is proportional to the expression value, computed as log2 [PZQ/Control]; red corresponds to positive log-ratios, *i*.*e*. these genes were up-regulated in paired females treated with PZQ when compared with the no-drug controls; grey corresponds to a gene present in the analysis but not differentially expressed. In humans, a number of gene homologs in this network encode proteins that are known drug targets and the corresponding drugs (Rx) are indicated.

Searching for *S*. *mansoni* genes whose human homolog gene products are known targets of drugs already used for other diseases, and analyzing the most significantly enriched network of genes affected by PZQ in opposite directions in paired and unpaired mature females (Fig 2), IPA pointed to three up-regulated genes in paired females, namely ATP1A2 (Smp_015020), MAPK (Smp_073490) and ERK (Smp_008260).

Regarding the SmATP1A2 human homolog, the hATP1A2 Na/K-ATPase enzyme is inhibited by four drugs (Fig 2): the first is OMP, a well-studied proton pump inhibitor that reduces gastric acid secretion [27,28] and also inhibits the Na/K-ATPase [29,30]. As described further below, OMP has been tested here in combination with PZQ. The other drugs targeting human ATP1A2 are digoxin, which plays an important role in the treatment of heart failure [31]; perphenazine, a first generation antipsychotic with a strong record of effectiveness and tolerability [32,33]; and the diuretic agent ethacrynic acid [34] (Fig 2). Regarding the SmMAPK human homolog, the hMAPK enzyme complex is inhibited by RO-3201195, a 5-aminopyrazol-4-yl ketone p38 inhibitor [35] and also inhibited by SCIO-469 [36], a drug that was well tolerated in phase I testing [37]. The latter also inhibits the human ERK enzyme complex (Fig 2).

Also of note in the most significantly enriched network (Fig 2) is the presence of genes encoding kinases, such as MAPK3 (Mitogen-activated protein kinase 3—Sjc_0044730), MAPK (Mitogen-activated protein kinase—Smp_073490), ERK (Extracellular signal-regulated kinase—Smp_008260), RPS6KA5 (Ribosomal protein S6 kinase, 90KDa, polypeptide 5—Smp_017900), as well as genes encoding signaling proteins such as ARF6 (ADP-ribosylation factor 6—Smp_000730), INPP1 (Inositol polyphosphate 1-phosphatase—Smp_017520), and GNA12 (Guanine nucleotide binding protein—Smp_074510). The MAPK pathway is evolutionarily well conserved and is involved in a variety of physiological processes including proliferation, differentiation, development, immune function, stress responses and apoptosis [38–40]. MAPK genes are essential for normal development and successful survival and reproduction of the schistosome parasite [41–43]. Previous studies have shown that, although PZQ induces severe damage to paired females, the vitelline gland and ovary are completely redeveloped and egg production is resumed under PZQ treatment [40]. Our finding that paired females showed a network of up-regulated kinase genes under PZQ treatment point to a possible explanation for the previously described redevelopment of the reproductive system.

The most significantly enriched network also includes up-regulated genes in paired females that encode tropomyosin (TPM1—identified in the array with a homolog from other species gi|256078894) [44], tubulin (TUBGCP3—Smp_159180) [45], crucial proteins in the cytoskeleton architecture. Ultrastructural analysis of *S*. *mansoni* worms has shown that damage to the basement membrane is characteristic of schistosomes exposed to sub-optimal dosages of PZQ [40]. Our results suggest that up-regulation of genes encoding cytoskeleton proteins could eventually compensate for cytoskeleton damage.

Regarding the transcriptional changes in *S*. *mansoni* paired females treated with PZQ, IPA pointed to five enriched networks (p-values from 10^−55^ to 10^−21^, genes listed in S5 Table) (Fig 1B) among the 486 genes whose expression levels were induced or repressed in this condition. Regarding unpaired mature females treated with PZQ, IPA analysis of the 1,434 affected genes also revealed five enriched networks (p-values from 10^−47^ to 10^−42^, genes in the networks are listed in S5 Table) (Fig 1C), irrespective of being affected in common with paired females.

### Genome-wide expression changes of paired males treated with PZQ compared with no-drug controls

In parallel, we assessed the global expression changes driven by PZQ in paired male worms. We identified 1,120 differentially expressed genes in paired males with significant changes (q-value < 0.05) when compared with the no-drug control (Table 2; S4 Table). IPA revealed five enriched networks of differentially expressed genes in paired males (p-values from 10^−43^ to 10^−29^) (Fig 1D). The list of genes in the networks is available in S5 Table.

**Table 2:** General information about gene expression in paired males exposed to PZQ compared with no-drug controls.

*S*. *mansoni* genes	Expressed genes ^a^	Paired Males		
		Diff. expressed ^b^	Down-regulated	Up-regulated
Detected on the microarrays	9,977	1,120	592	528
Predicted in the genome	7,039	709	378	331
Not predicted in the genome ^c^	2,938	411	214	197

^a^ Number of genes expressed in at least one condition, *i*.*e*. in control or in treated paired male adult worms.

^b^ q-value < 5%

^c^ Either predicted in *S*. *japonicum*, present in other species, or detected only in *S*. *mansoni* without close homologs in other species (no match).

**Fig 3:**
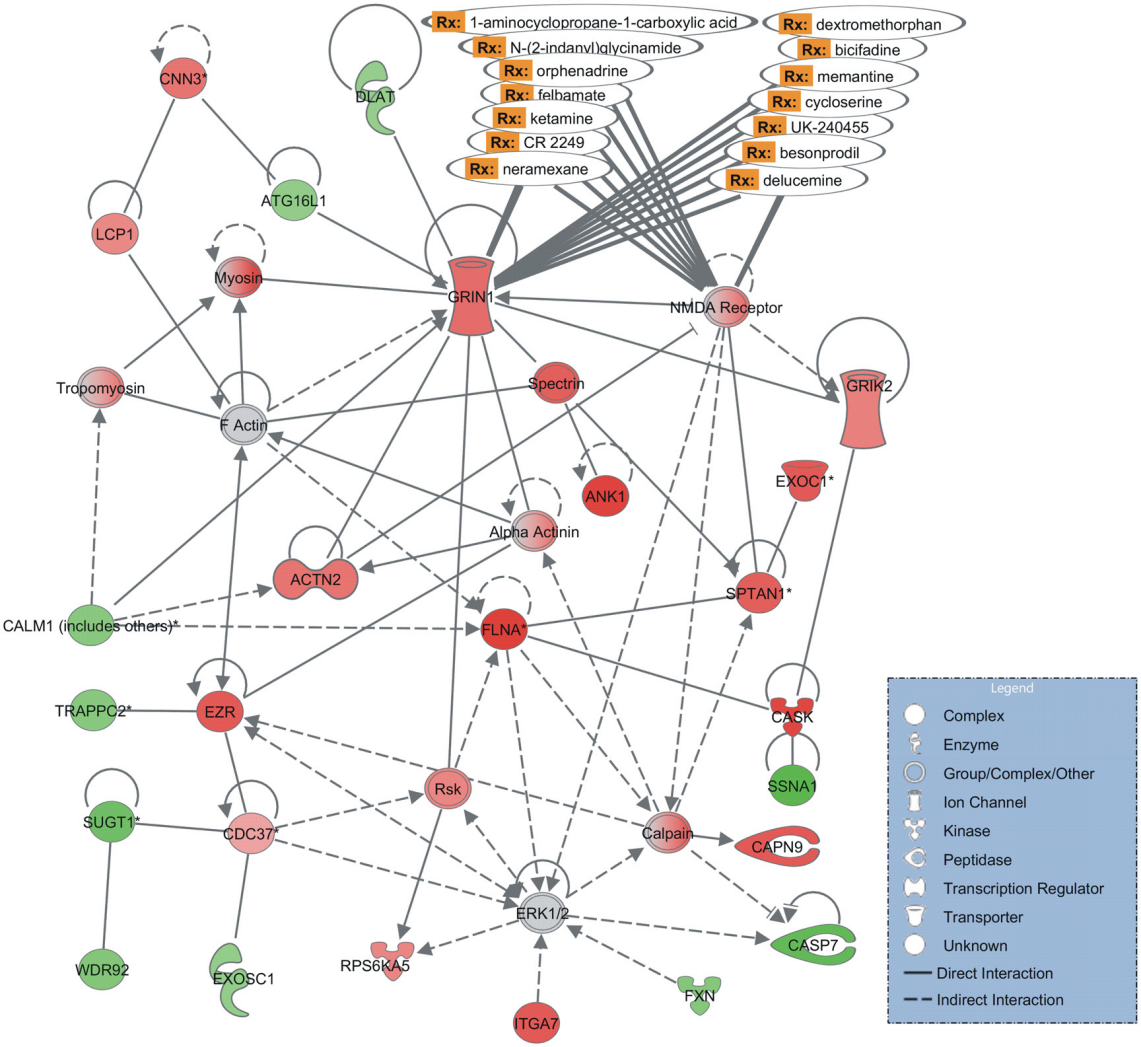
Most significantly enriched interaction network of differentially expressed genes in PZQ-treated paired males. The gene interaction network is related to cellular assembly and organization, cell-to-cell signaling and interaction. The shapes of elements correspond to different types of molecules, as indicated in the inset box. Arrows indicate the relationship between the elements: dashed or solid lines indicate indirect or direct interactions, respectively. The color intensity is proportional to expression value, computed as log2 [PZQ/Control]; red corresponds to positive log-ratios, *i*.*e*. genes up-regulated in paired males treated with PZQ when compared with no-drug controls, green corresponds to negative log-ratios, *i*.*e*. down-regulated genes, and grey corresponds to those genes present in the analysis but not differentially expressed. Two genes from this network encode human proteins that are known drug targets and the corresponding drugs (Rx) are indicated.

The first most significantly enriched network (p-value = 10^−43^) is associated to functions such as cellular assembly and organization, cell-to-cell signaling and interaction (Fig 3). The schistosome tegument provides structural and functional elements for nutrient uptake and physical and immunological protection [46–48]. In this network we identified tegument genes, such as actin-2 (Smp_034550) and alpha actin (Smp_034550), tropomyosin (Smp_031770), and myosin (identified in the array with a homolog from other species gi|547978), which encode proteins that participate in muscle contraction, and are also part of the cytoskeleton. These affected genes are in line with the physiological effects of PZQ described in the literature: it is well-known that PZQ causes early spastic paralysis of the worm musculature, vacuolization at the base of the tegumental syncytium, and blebbing on the surface [49]. Morphological changes are accompanied by an increased exposure of parasite antigens on the worm surface [50]. It is also known that tegument genes up-regulation is an early response to PZQ of male worms, possibly reflecting tegument stress [48].

We identified two glutamate receptor genes, GRIN1 (Smp_126350) and NMDA receptor (Smp_126350), as up-regulated and present in the enriched network of genes affected by PZQ in paired males (Fig 3). The proteins encoded by these genes may be related to the L-glutamate-induced contractions observed in isolated *S*. *mansoni* muscle fibers [51]. It is noteworthy that a new glutamate receptor SmGluR, belonging to the G protein-coupled receptor (GPCR) superfamily has been characterized in neurons and the female reproductive tract of *S*. *mansoni* worms [52]. Also, the presence of ivermectin-insensitive glutamate-gated chloride channels has been described in *S*. *mansoni* [53]. Glutamate receptors are particularly promising drug targets because of their involvement in fast excitatory transmission [54]. Many neuroactive drugs under development for treatment of human diseases target glutamate receptors [55–57] and the mainstay of nematode control, ivermectin, works through a glutamate receptor [58]. PZQ induces muscular contractures that lead to tegumental rupture and death [8]. It is conceivable that a L-glutamate receptor agonist could enhance parasite muscle contraction and amplify PZQ effect.

### Common gene targets in female and male worms can point to important drug candidates to be tested as possible schistosomicides

Despite the highly divergent transcriptional patterns between male and female worms [59], we found 48 overlapping genes that were affected by PZQ in both paired males and females (Fig B (upper panel) in S1 Text). Notably, 85% of these genes showed a similar pattern of expression in the two genders (either up-regulated in both, or down-regulated in both, upon treatment) (Fig B (lower panel) in S1 Text; S4 Table), probably resulting from gender-independent effects of PZQ. The finding of a predominantly similar pattern of expression in the two genders, described here, is in contrast with the findings from a recent comparison of gene transcription in worms exposed to PZQ *in vitro* [60]; the difference is possibly due to the fact that the authors used a 10-fold higher concentration of PZQ [60] than the one used here. Hines-Kay and co-workers [60] compared the effect of PZQ on gene expression between adult *S*. *mansoni* male and female parasites and showed that, among the genes that were affected by PZQ in both males and females, only 20% were changed in the same direction (either up or down) in both sexes. The different patterns of expression changes between the two works are in line with the known differential susceptibility of the two genders to the different tested concentrations of PZQ.

You and co-workers [48] have investigated the expression changes in *S*. *japonicum* recovered from infected mice treated with sublethal doses of PZQ, and have highlighted CamKII, a putative calcium/calmodulin-dependent protein kinase type II delta chain, as the leading candidate among the genes affected by PZQ [48]. In our *in vitro* assay, *S*. *mansoni* CamKII (Smp_011660) was not detected as differentially expressed either in females or males. Nonetheless, five other genes belonging to the calcium-signaling pathway and identified as differentially expressed by You and co-workers [48] were also detected as significantly changed in our conditions. Three calcium-transporters, namely PMCA, the plasma membrane calcium-transporting ATPase (Smp_176130), SERCA, the sarcoplasmic/endoplasmic reticulum calcium-transport ATPase (Smp_136710), and RYR, the ryanodine receptor (Smp_163570), were up-regulated in paired males in both *S*. *mansoni* and *japonicum* datasets. The other two genes were CaN and PKC. CaN, the protein phosphatase-2b (Smp_126260), that was up-regulated in unpaired mature females in our dataset as well as in *S*. *japonicum* females [48]. PKC, the serine/threonine protein kinase (Smp_176360), was down-regulated in unpaired mature females as well as in *S*. *japonicum* females [48].

**Fig 4:**
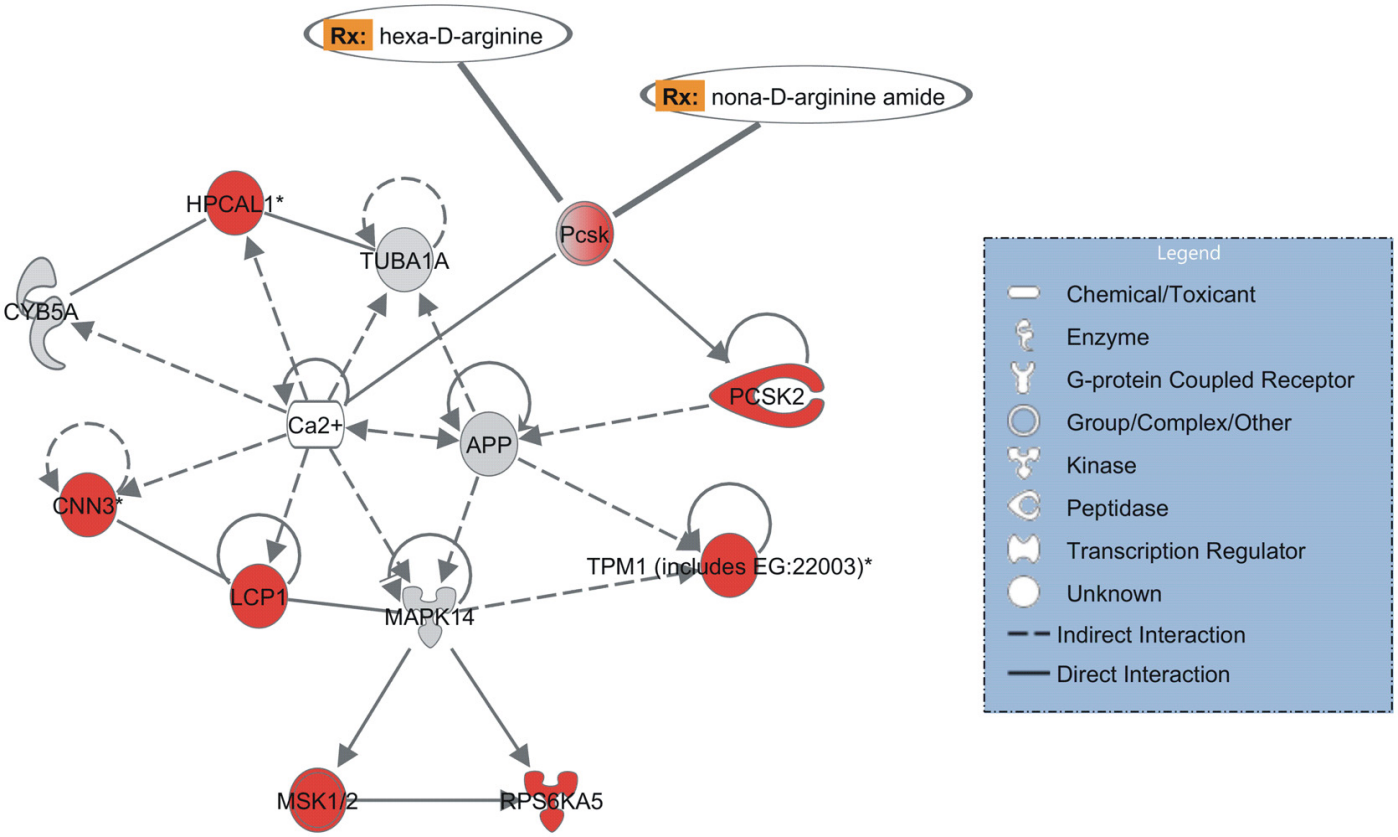
Enriched gene interaction network detected with similar expression pattern in PZQ-treated paired males and females. The gene interaction network is related to carbohydrate metabolism, molecular transport and small molecule biochemistry. The shapes of elements correspond to different types of molecules, as indicated in the inset box. Arrows indicate the relationship between the elements: dashed or solid lines indicate indirect or direct interactions, respectively. The color intensity is proportional to expression value, computed as log2 [PZQ/Control]; red corresponds to positive log-ratios, *i*.*e*. genes up-regulated in paired males and females treated with PZQ when compared with their respective no-drug controls, grey corresponds to those genes present in the analysis but not differentially expressed. In humans, one gene homolog in this network encodes a protein that is a known drug target and the corresponding drugs (Rx) are indicated.

Functional analysis revealed one interaction network that was significantly enriched (p-value = 10^−17^) with genes whose expression was equally affected (predominantly induced) in paired males and females by PZQ treatment (Fig 1E); this network is associated with carbohydrate metabolism, molecular transport, and small molecule biochemistry. Interestingly, calcium ion is a ligand that is in the center of this network (Fig 4), regulating the proteins related to muscle contraction encoded by the genes present in the network (see list of genes in S5 Table). PZQ produces a well-documented effect on intracellular Ca2+ levels in adult schistosomes [61]. HPCAL1 (Putative neuronal calcium sensor—Smp_068510) and CNN3 (Calponin—Smp_078690), both implicated in the regulation and modulation of smooth muscle contraction, were up-regulated in *S*. *mansoni* males and females exposed to the sublethal dose of PZQ.

It is noteworthy that the human homolog of Pcsk proprotein convertase gene, present in the enriched network described above (Fig 4) is a drug target. Our results supports findings of a recent comparative chemogenomics strategy that predict the protein encoded by Smp_077980 as one out of 35 potential drug targets in *S*. *mansoni* [62]. Because of the role played by these protein convertases in humans, Pcsks are now considered to be attractive targets for the development of powerful novel therapeutics [63]. Two synthetic peptides, hexa-D-arginine and nona-D-arginine amide, have been investigated as Pcsk inhibitors in therapeutic strategies against anthrax [64]. As Pcsk is a common target for female and male worms, and this gene is up-regulated in worms under the physiological mated condition, these Pcsk inhibitors could be important drug candidates to be tested *in vitro* and also in the mouse model as possible schistosomicidal combination drugs along with PZQ.

### Validation of differentially expressed protein-coding genes by Reverse Transcription-quantitative Real Time PCR

We selected 9 differentially expressed genes affected by PZQ in both paired and unpaired mature females, when compared with their no-drug respective controls, for validation of expression changes by Reverse Transcription—Quantitative Real Time PCR (RT-qPCR). The nine selected genes were: ATP1A2 (Smp_015020—ATPase, Na+/K+ transporting, alpha 2 polypeptide), ARF6 (Smp_000730—ADP-ribosylation factor 6), EXOC2 (Smp_131960—exocyst complex component 2), EXOC5 (Smp_148070—exocyst complex component 5), GNA12 (Smp_074510—guanine nucleotide binding protein (G protein) alpha 12), INPP1 (Smp_017520—inositol polyphosphate-1-phosphatase), MAPK3 (Sjc_0044730—mitogen-activated protein kinase 3), RPS6A5 (Smp_017900—ribosomal protein S6 kinase, 90kDa, polypeptide 5), TUBGCP3 (Smp_159180—tubulin, gamma complex associated protein 3).

**Fig 5:**
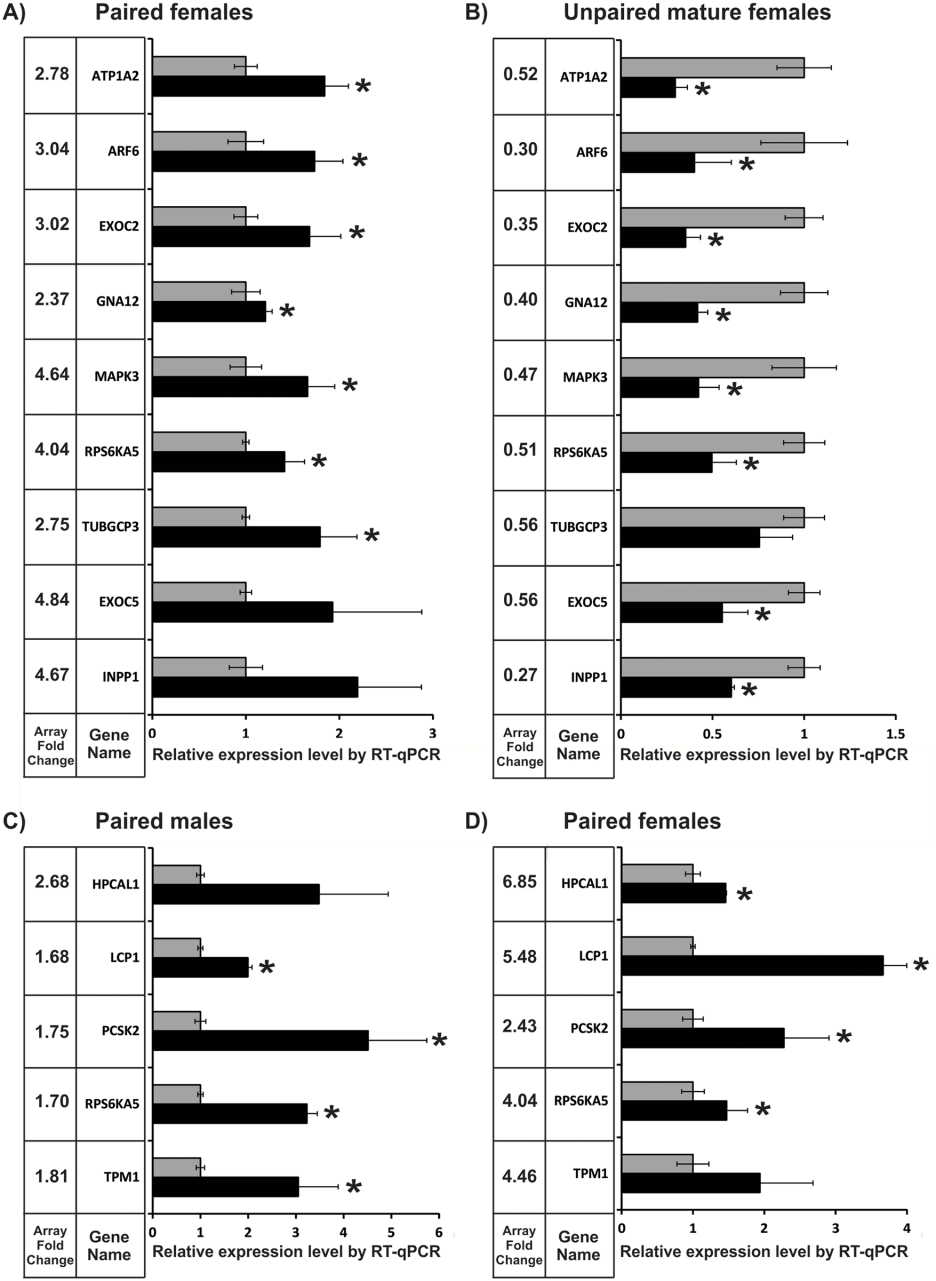
Validation by RT-qPCR of expression changes induced by PZQ treatment in *S*. *mansoni* adult worms. The expression levels of genes were measured by RT-qPCR in parasites treated with PZQ (black bars) relative to the levels in the respective no-drug control parasites (gray bars). Genes were measured (A) in paired females; (B) in unpaired mature females; (C) in paired males; and (D) in paired females. Genes with significant difference (t-test, p < 0.05) are marked with asterisk. Next to each gene name, the gene expression fold-change induced by PZQ and detected by microarray is shown for comparison.

Fig 5A shows that for paired females seven out of the nine genes were successfully validated by RT-qPCR, whereas no changes in expression were detected for EXOC5 and INPP1. Fig 5B shows that eight out of the nine selected genes were successfully validated for unpaired mature females, with statistical significance. In addition, the direction of change in expression was in agreement between both microarray and RT-qPCR for all tested genes, i.e. the genes were up-regulated in paired females when compared with no-drug controls, and down-regulated in unpaired mature females relative to controls both by microarray and RT-qPCR.

We further selected five genes for RT-qPCR validation that were affected by PZQ in paired males and females, when compared with their no-drug respective controls: PCSK2 (Smp_077980—proprotein convertase subtilisin/kexin type 2), TPM1 (identified in the array with a homolog from other species gi|256078894—tropomyosin 1), LCP1 (Smp_037230—lymphocyte cytosolic protein 1), RPS6KA5 (Smp_017900—ribosomal protein S6 kinase, 90kDa, polypeptide 5), HPCAL1 (Smp_068510—hippocalcin-like 1).

Fig 5C shows that four out of the five selected genes (80%) were successfully validated by RT-qPCR for paired males with statistical significance, while HPCAL1 change was not significant. Fig 5D shows again that four out of the five selected genes (80%) were successfully validated for paired females, while TPM1 change was not significant.

Selection of genes for validation was based on the fact that most of these genes were highlighted by the functional analyses mentioned above. Twenty-three out of the twenty-eight conditions (82%) that have been tested (Fig 5), among the 13 different protein coding genes that were selected for validation by RT-qPCR, had significant expression changes confirmed. Overall, the Spearman correlation between microarray data and RT-qPCR was 0.53 with a p-value = 0.004. We consider it to be a successful validation, especially if we note that none of the directions of change in the microarray and RT-qPCR data showed a conflicting result; it is already known that both experimental techniques, microarray and qPCR have inherent pitfalls that affect quantification and cannot be fully controlled [65].

### Effect of OMP on adult schistosomes treated with PZQ

We based our choice of OMP on the IPA results (Fig 2), which show a gene interaction network, enriched with PZQ-induced differentially expressed genes in females, that comprises three *S*. *mansoni* genes whose human homologs encode proteins that are known targets of different drugs (Fig 2) already tested in humans for other diseases. For the drug synergy test between PZQ and a possible new schistosome inhibitor, we chose OMP (Fig 2), a drug widely prescribed for children and adults that targets the human ATP1A2 enzyme. OMP is one of the most widely prescribed drugs for peptic ulcer treatment, has an FDA-approved generic version, is available over-the-counter in some countries, and is on the World Health Organization's List of Essential Medicines. OMP inhibits the human (H^+^/K^+^)-ATPase gastric proton pump, which may be responsible for acid secretion [28]; it has also been shown to inhibit the kidney and brain (Na^+^/K^+^)-ATPase [29,30].

Another criterion to choose OMP was that the expression of SmATP1A2 was significantly up-regulated in paired females when the parasites were exposed to PZQ (Fig 2). Although the microarray expression analysis of males treated with PZQ did not show ATP1A2 as a differentially expressed gene (with the significance cut off that we selected for the array data), RT-qPCR has clearly detected ATP1A2 as significantly up-regulated in male worms (Fig C (upper panel) in S1 Text).

In addition, OMP has already been shown to be synergistic with the first-line treatment drug quinine against the *Plasmodium falciparum* protozoan parasite [66]. In that work, EC50 for OMP alone was determined to be 14 to 36 μM [66], and we therefore decided to choose a 2-fold higher concentration of OMP (25 μg/ml, 72 μM) to be used in our experiments, with a more complex platyhelminth.

We first determined a sublethal concentration of PZQ that killed only a very small percentage of adult worms; because males are more susceptible to PZQ than females [9], we determined a separate specific sublethal concentration for each gender (150 ng/ml PZQ for males and 532 ng/ml PZQ for females) (see Methods).

Next, the possible synergistic effect of PZQ and OMP was assayed by looking at the survival of *S*. *mansoni* paired adult worms incubated *in vitro* with PZQ or OMP alone and in combination. The parasites were monitored for survival over a period of 120 h. The negative control corresponded to adult worms assayed in 1.5% DMSO.

**Fig 6:**
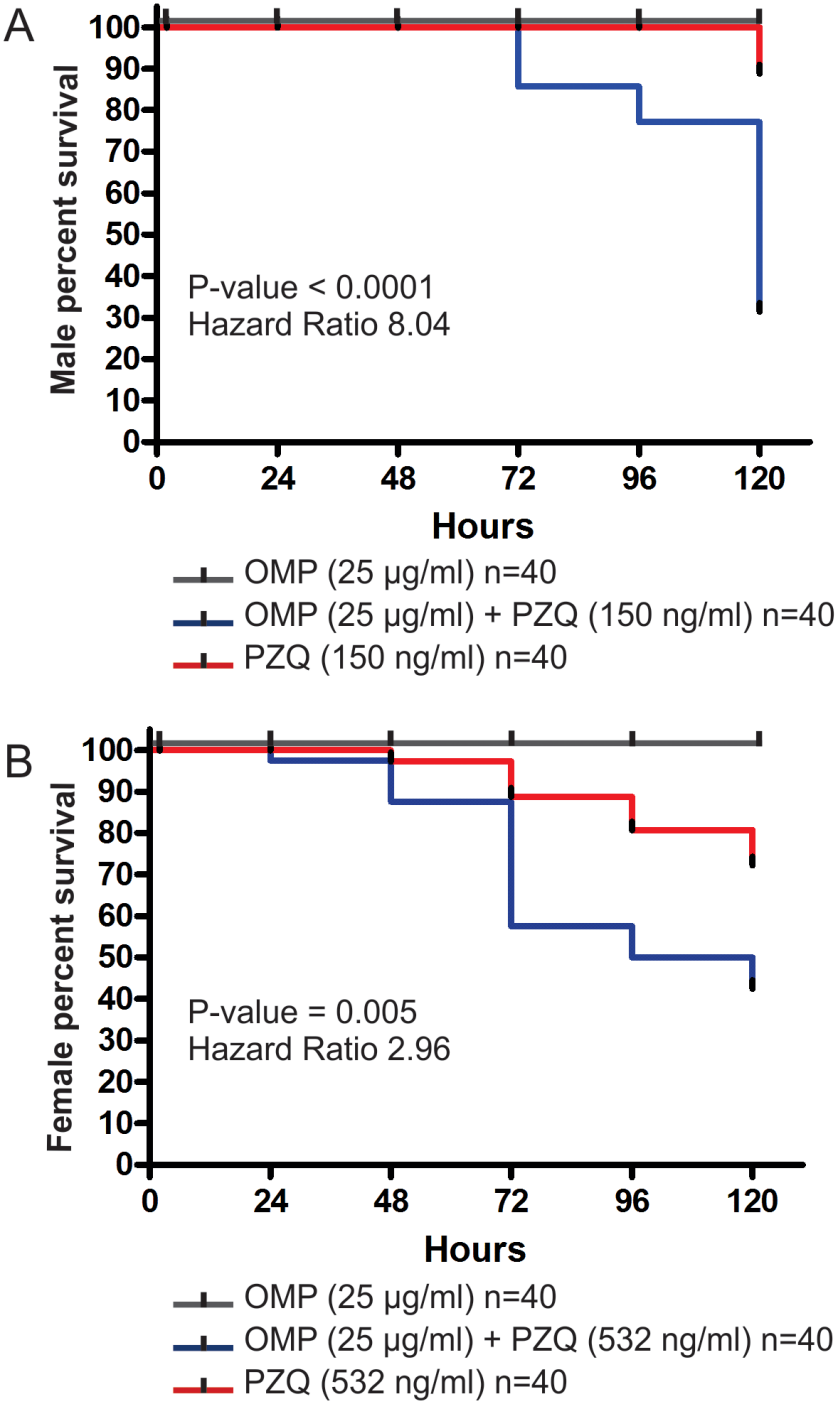
Kaplan-Meier survival curves for adult *S*. *mansoni* worms treated *in vitro* with PZQ+OMP, PZQ or OMP. Adult worm couples were treated with a synergistic combination of PZQ+OMP (blue line), with PZQ alone (red line) or with OMP alone (black line). The assays were performed with paired adult worm couples (20 couples for each of the three conditions in each of the two biological replicates) and the fraction of surviving worms was recorded after 2, 24, 48, 72, 96 and 120 hours; the Kaplan-Meier survival curve was calculated using all the events of the two biological replicas together, and the Log-rank Mantel-Cox statistical test was used to calculate the significance of the Hazard Ratio between OMP+PZQ and PZQ alone. The effect of the synergistic combination was analyzed for each gender separately: (A) for scoring males percent survival, couples were incubated in the presence of 150 ng/ml PZQ, with or without 25 μg/ml OMP, or in the presence of 25 μg/ml OMP alone; and (B) for scoring females percent survival, couples were incubated in the presence of 532 ng/ml PZQ, with or without 25 μg/ml OMP, or in the presence of 25 μg/ml OMP alone.

To obtain a statistical estimate of the significance of the PZQ+OMP effect, we performed a Kaplan-Meier survival analysis of male or female adult worms treated with PZQ versus PZQ+OMP, using all the events of the two biological replicas in the analysis. Fig 6A shows the Kaplan-Meier curve of male survival, which revealed an 8-fold statistical increase in male death (*p*-value < 0.0001) for paired worms treated with the synergistic combination of a sublethal dose of PZQ (150 ng/ml) + 25 μg/ml OMP (Fig 6A, blue line), when compared with male death in worm pairs treated with the sublethal dose of PZQ alone (Fig 6A, red line). For the females survival assay, worm couples were treated with a synergistic combination of PZQ (532 ng/ml) + 25 μg/ml OMP, which caused a 2.98-fold statistical increase in female death (*p*-value = 0.005) (Fig 6B, blue line) when compared with the female death in worm pairs treated with PZQ (532 ng/ml) alone (Fig 6B, red line). In contrast, male and female worms remained viable until the end of the incubation period (120 h) in the groups treated with 25 μg/ml OMP alone (Fig 6A and 6B, black lines), as well as in the no-drugs negative control group.

The above results show that OMP at the tested dose does not kill viable adult (male or female) worms, just affecting PZQ-stressed parasites. This is in contrast to the effect of OMP on protozoans [67–70], which are killed by OMP alone. In fact, Jiang et al. [69] have shown that in *Leishmania donovani* the antiparasitic action of OMP is due to its inhibition of the P-type K/H-ATPase on the surface membrane. Here, the PZQ+OMP synergistic effect against *S*. *mansoni* was obtained with a dose of 72 μM OMP (25 μg/ml) that is similar to the EC50 of 76 μM OMP against *P*. *falciparum* [68]. It seems that the synergistic action of OMP+PZQ against PZQ-stressed *S*. *mansoni* could be dependent on the increased expression of the ATP1A2 Na/K-ATPase caused by PZQ. This would suggest that OMP is counteracting the increased Na/K-ATPase activity in PZQ-stressed *S*. *mansoni* parasites. On the other hand, it is known that OMP also targets the ATP-dependent efflux transporter P-glycoprotein [71], a member of the ABC multidrug transporter family. In *S*. *mansoni* one member of this group was identified as SMDR2 (*S*. *mansoni* Multidrug Resistance 2) encoding a Pgp-like protein [72]. We detected the SMDR2 gene as up-regulated in paired females treated with PZQ, while in unpaired mature females the opposite expression change was observed. It has been shown that SMDR2 activity is inhibited by PZQ in a recombinant protein assay [73], and that inhibition by the specific drug tariquidar or knockdown of SMDR2 P-glycoprotein enhances PZQ activity [74] by a mechanism that involves an increased retention of labeled-PZQ inside the worms [74]. Therefore, there is a possibility that OMP would similarly act through inhibition of the P-glycoprotein in PZQ-stressed parasites, to increase the retention of PZQ and parasite susceptibility. Further studies are warranted, such as those for measuring the retention of labeled-PZQ in the presence of OMP, or for measuring the effect of OMP upon knockdown of ATP1A2 Na/K-ATPase or SMDR2 P-glycoprotein, to determine whether OMP is acting through either or both possible mechanisms.

### Effect of OMP on schistosomula treated with PZQ

We also investigated the effects of OMP or OMP+PZQ for the ability to affect schistosome larvae maintained in culture; 3-hour-old schistosomula were cultured *in vitro* in the presence of the same concentrations of OMP or OMP+PZQ used for the adult worm couples test, mobility and death were monitored, and a typical experiment is shown in Fig D in S1 Text. Both OMP and PZQ were non-toxic for schistosomula over a period of 7 days. After 24- hour incubation the schistosomula were alive and morphologically normal (Fig D in S1 Text) and no red fluorescence signal (no dead larvae) could be detected under all concentrations of PZQ or PZQ+OMP tested. On day 7 a slight contraction was observed, although no opaque worms or immobile larvae were observed, and no red fluorescence signal (no dead larvae) could be detected (Fig D in S1 Text). The schistosomula in the negative control group also remained viable until the end of the incubation period. In order to have a positive control, we incubated schistosomula at a very high PZQ concentration (10 μg/ml PZQ, 20 to 60 X higher than in the test experiments) and found that approximately 30% of the larvae were dead after incubation for 7 days (Fig E in S1 Text).

To further characterize the effect of OMP+PZQ on the gene expression pattern of schistosomula, we measured by RT-qPCR the expression of a selected set of 9 genes on schistosome larvae exposed to 532 ng/ml PZQ for 24 hours. We selected the same 9 genes that were detected as differentially expressed in mature adult worms (males and females), including ATP1A2 (Smp_015020). Fig C (lower panel) in S1 Text shows that no statistically significant expression changes were detected in PZQ-treated schistosomula for eight of the tested genes, including ATP1A2, MAPK3, ARF6, EXOC2, GNA12, RPS6KA5, TUBGCP3 and INPP1. Only EXOC5 out of the nine genes had a statistically significant increase in gene expression after 24-hour treatment of schistosomula with 532 ng/ml PZQ.

As already discussed in the previous section, the ATP1A2 gene product is one of the possible drug targets of OMP. In adult worms, ATP1A2 expression was increased upon exposure of the worms to PZQ, and it is conceivable that an increase in ATP1A2 activity is part of the survival response in these adult worms under PZQ stress. Hence, inhibition of ATP1A2 by OMP in the presence of PZQ would increase the mortality of adult worms. Once no change in expression of ATP1A2 was observed in schistosomula in the presence of PZQ, a synergistic effect of OMP+PZQ would not be expected in schistosomula.

In this respect, it is worth mentioning that juvenile schistosomes (3–4 weeks post-infection), which are normally refractory to 2 μM PZQ, became paralyzed when ABC multidrug transporter inhibitors were added in combination with PZQ [74]. In this scenario, a critical experiment would be to test the effect of OMP+PZQ on PZQ-refractory juvenile worms, and to measure the expression of the ATP1A2 gene under the same conditions.

### Conclusions

Taken together, our results show a global PZQ-driven transcriptional alteration related to gender and to the mating status of *S*. *mansoni* mature females. Our data help to understand the molecular basis of the differences in susceptibility to PZQ between males and paired and unpaired mature females, and therefore differences in the possible mechanisms of drug action. Using functional analysis of gene interaction networks we were able to identify genes affected by PZQ whose human homolog encoded proteins are known targets of a number of drugs already tested in humans for other disease conditions. Among these drugs was OMP, which proved to have a synergistic effect against *S*. *mansoni* when tested *in vitro* in combination with PZQ. Consequently, this study is a proof of concept that integrative network analysis is an important approach to identify synergistic drugs. Repurposing of existing drugs for schistosomiasis is appealing [26], as such a strategy should be far cheaper than developing a new compound from scratch [75]. Additional studies are necessary to elucidate the effect of the combination of PZQ and OMP against *S*. *mansoni* adult worms *in vivo*.

## Supporting Information

S1 Text Comprises the following supplementary figures. Fig A—Heat map of differentially expressed genes detected in paired and unpaired mature *S*. *mansoni* females; Fig B—Heat map of differentially expressed genes detected in paired male and female adult worms; Fig C—Measurement by Reverse Transcription Real Time PCR of the expression changes induced by PZQ treatment in *S*. *mansoni*; Fig D—Schistosomula viability after 1 day and 7 days treatment with OMP or OMP+PZQ; Fig E—Schistosomula viability in a positive control assay with an extremely high concentration of PZQ.
(PDF)

S1 Table Complete list of selected probes (marked "YES, To be used in expression analysis") and their corresponding *S*. *mansoni* gene annotations. These probes were selected because they showed the smallest coefficient of variation of probe intensity, among the different probes for the same Smp gene on the microarray, when analyzing the replicas of almost all previously published experiments using this array.
(XLS)

S2 Table Complete list of the GI number of each human putative homolog gene associated to the corresponding *S*. *mansoni* probe on the array. All probes are in the sense strand and represent *S*. *mansoni* protein-coding genes that were associated to putative human homolog genes.
(XLSX)

S3 Table List of oligonucleotides used in Real Time RT-PCR. (XLSX)

S4 Table List of the S. mansoni differentially expressed genes detected in paired females only, in unpaired mature females only, simultaneously in paired and unpaired mature females, in paired males only, and simultaneously in paired female and paired male worms, all of them treated with PZQ for 16 hours. (XLSX)

S5 Table List of differentially expressed genes that comprise the most significantly enriched gene interaction networks in the following comparisons: genes simultaneously changed in paired and unpaired mature females treated with PZQ; genes in paired female worms treated with PZQ compared with the no-drug controls; genes in unpaired mature female worms treated with PZQ compared with the no-drug controls; genes in paired male worms treated with PZQ compared with the no-drug controls; genes simultaneously changed in paired males and females treated with PZQ. (XLSX)
