# Supplementary material for: Synergy of Omeprazole and Praziquantel In Vitro Treatment against Schistosoma mansoni Adult Worms
Source: PLoS Negl Trop Dis. 2015 Sep 24;9(9):e0004086. doi: 10.1371/journal.pntd.0004086 (PMC4581627; doi:10.1371/journal.pntd.0004086)
Supplement: S1 Text — Fig A—Heat map of differentially expressed genes detected in paired and unpaired mature S. mansoni females; Fig B—Heat map of differentially expressed genes detected in paired male and female adult worms; Fig C—Measurement by Reverse Transcription Real Time PCR of the expression changes induced by PZQ treatment in S. mansoni; Fig D—Schistosomula viability after 1 day and 7 days treatment with OMP or OMP+PZQ; Fig E—Schistosomula viability in a positive control assay with an extremely high concentration of PZQ. (PDF) [file pntd.0004086.s001.pdf]

Supporting Information

Synergy of Omeprazole and Praziquantel *in vitro* Treatment Against *Schistosoma mansoni* Adult Worms

Giulliana T. Almeida[1¶], Regina C. G. Lage[2¶], Leticia Anderson[1], Thiago M. Venancio[1#a], Helder I Nakaya[1], Patrícia A. Miyasato[3], Henrique K. Rofatto[3], Adhemar Zerlotini[2], Eliana Nakano[3], Guilherme Oliveira[2,4], Sergio Verjovski-Almeida[1,3*]

1 - Departamento de Bioquímica, Instituto de Química, Universidade de São Paulo, 05508-900 São Paulo, SP, Brazil, 2 – Genomics and Computational Biology Group, Centro de Pesquisas René Rachou - FIOCRUZ, 30190-002 Belo Horizonte, MG, Brazil, 3 - Instituto Butantan, 05503-900 São Paulo, SP, Brazil, 4 – Vale Technology Institute, Rua Boaventura da Silva 955, 66055-090 Belém, PA, Brazil.

[#a] Current address: Laboratório de Química e Função de Proteínas e Peptídeos, Centro de Biociências e Biotecnologia, Universidade Estadual do Norte Fluminense Darcy Ribeiro, Rio de Janeiro, Brazil.

* Corresponding author e-mail: verjo@iq.usp.br

¶ These authors contributed equally to this work

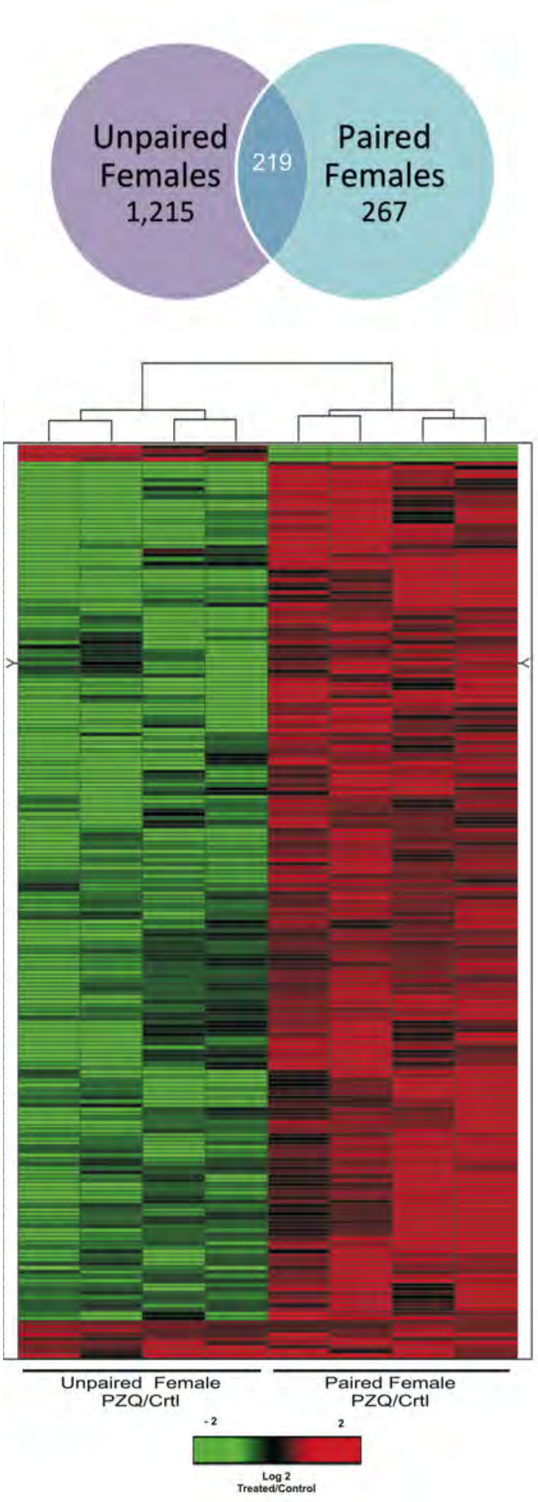

Fig A – Differentially expressed genes detected in paired and unpaired mature *S. mansoni* females. **(Upper)** Venn diagram of the genes affect by PZQ in both paired and unpaired mature females, when compared with their respective no-drug controls. **(Lower)** Heat map of 219 differentially expressed genes affect by PZQ in both experimental conditions, namely paired females and unpaired mature females.

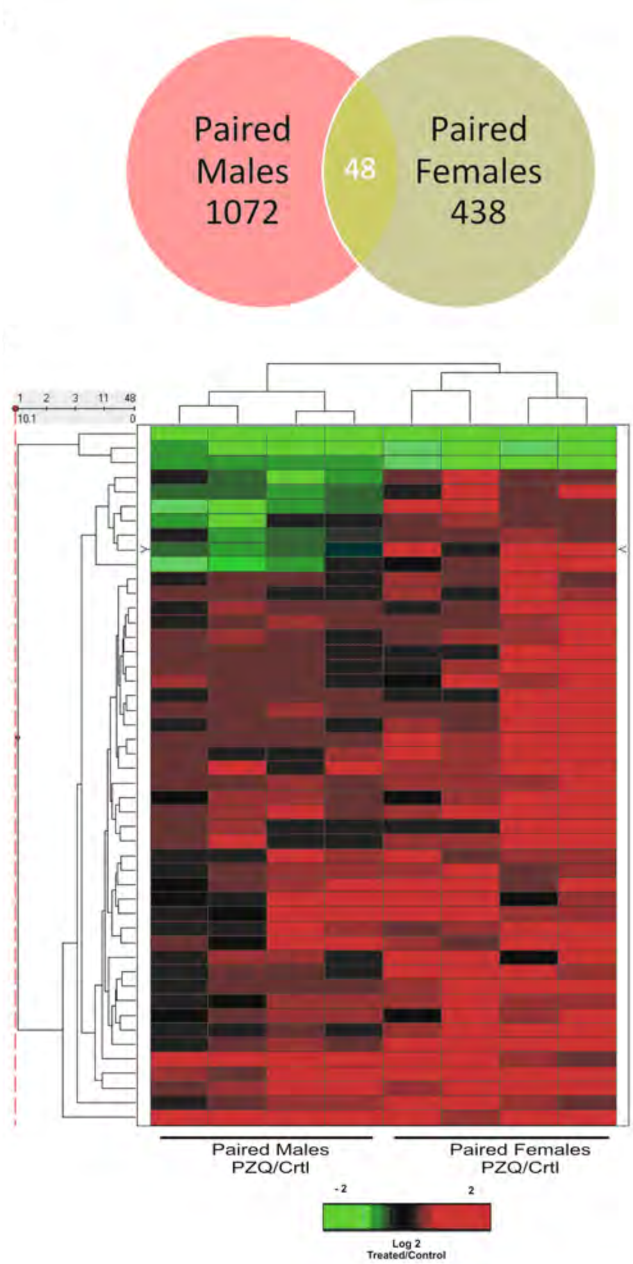

Fig B – Differentially expressed genes detected in paired male and female adult worms. **(Upper)** Venn diagram of the genes affect by PZQ in paired males and paired females, when compared with their respective no-drug controls. **(Lower)** Heat map of 48 differentially expressed genes affected in both experimental conditions (paired males and females).

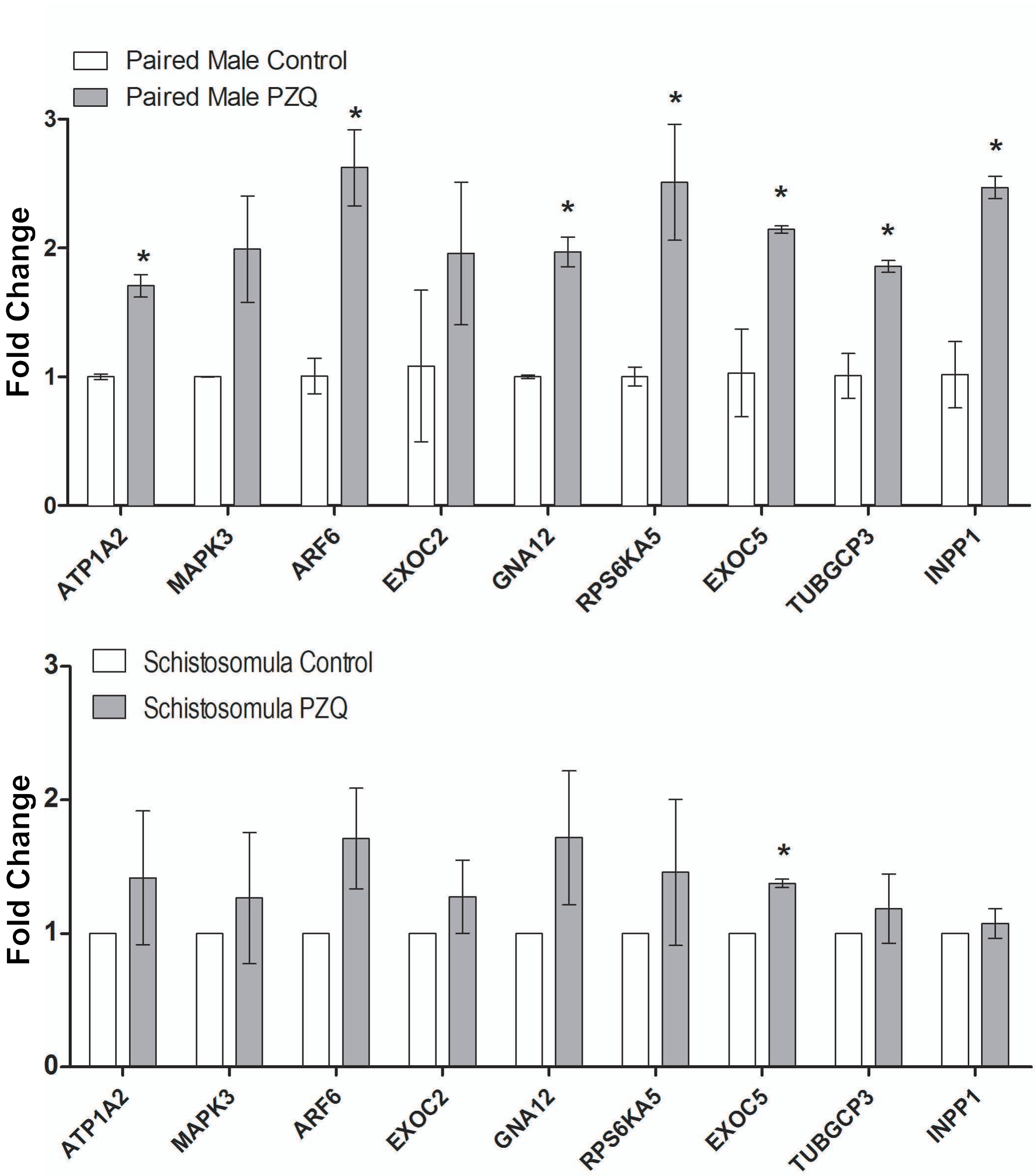

Fig C - Measurement by Reverse Transcription Real Time PCR of the expression changes induced by PZQ treatment in *S. mansoni*. (Upper) Genes affected by PZQ in paired males, when compared to their non-treated control. **(Lower)** Genes affected by PZQ in 3h old schistosomula, when compared with their non-treated controls. A t-test was used to calculate the significance of the difference between PZQ and Control (n = 2 biological replicates, with 3 technical replicates each); genes with significant difference ($p < 0.05$) are marked with asterisks.

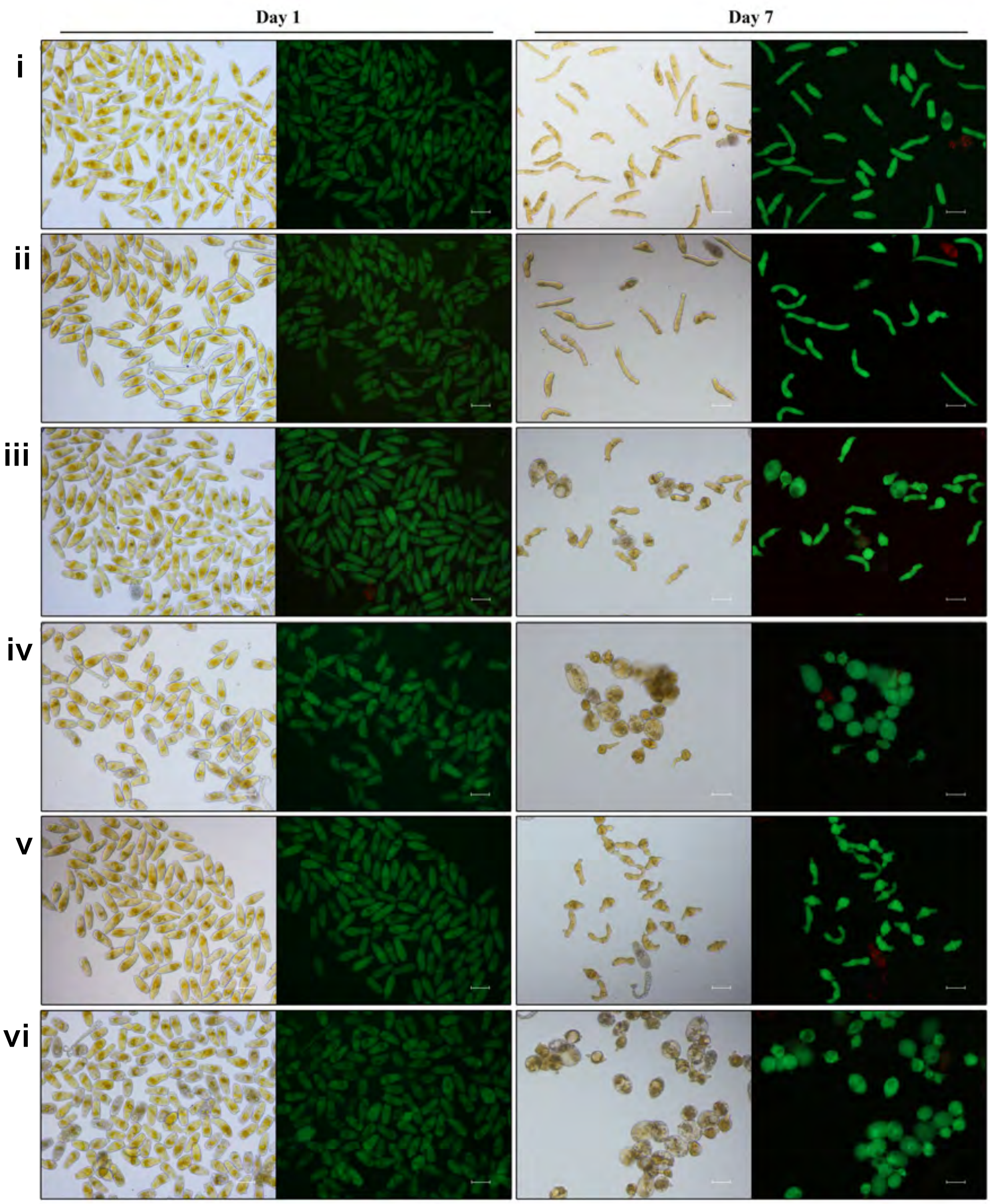

Fig D - Schistosomula viability after 1 day and 7 days treatment with OMP or OMP+PZQ. Schistosomula were photographed on day 1 (columns 1 and 2) or on day 7 (columns 3 and 4) after treatment. Columns 1 and 3 show light microscopy images; columns 2 and 4 show green fluorescence of FDA from live cells, and red fluorescence of PI from dead cells. (i) negative control with no drugs; (ii) 25 μg/ml OMP; (iii) 150 or (iv) 532 ng/ml PZQ; (v) 25 μg/ml OMP + 150 ng/ml PZQ; and (vi) 25 μg/ml OMP + 532 ng/ml PZQ. The calibration bar shows 100 μm.

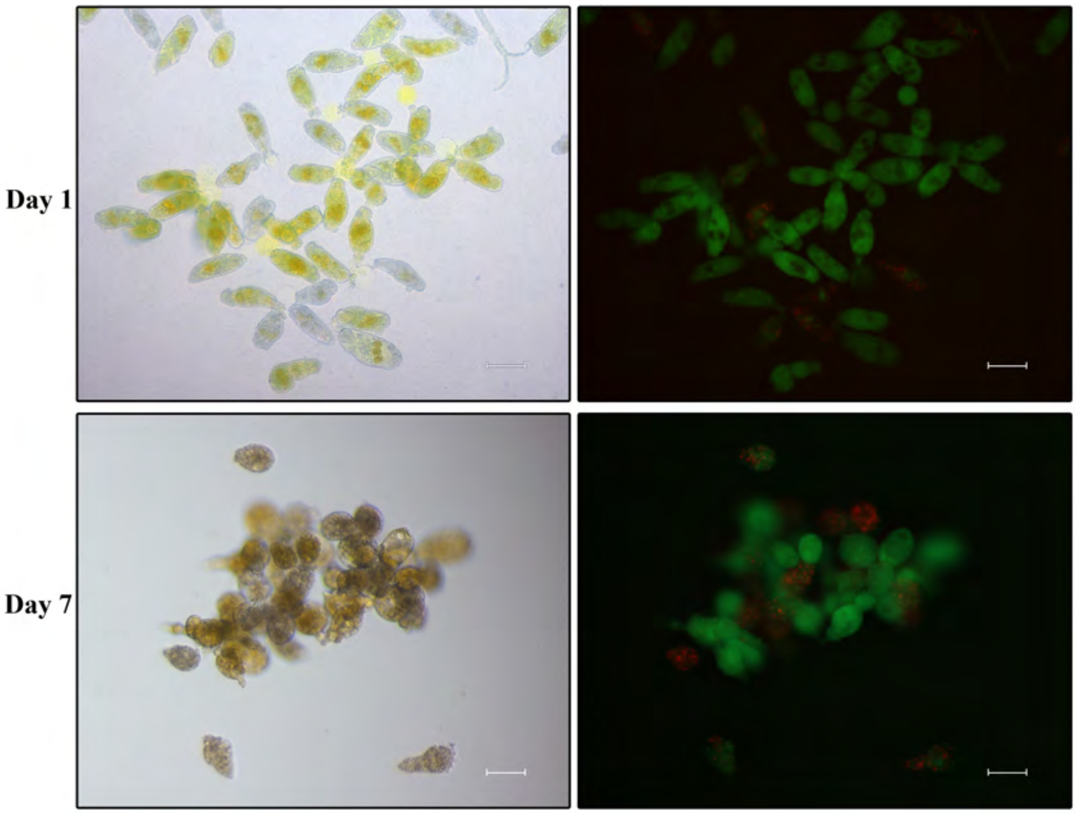

Fig E - Schistosomula viability in a positive control assay with an extremely high concentration of PZQ. Schistosomula were treated with 10 µg/ml PZQ (20 to 60 X higher than the sub-lethal test concentrations of the other experiments) and photographed after 1 day (top panels) and 7 days (bottom panels). On day 1 all schistosomula remained viable. Approximately 30% of the larvae were dead on day 7. **(Left)** Light microscopy at days 1 and 7. **(Right)** Green fluorescence of FDA from live cells, and red fluorescence of PI from dead cells, at days 1 and 7. The calibration bars show 100 µm.
